# Nonlinear self-calibrated spectrometer with single GeSe-InSe heterojunction device

**DOI:** 10.1126/sciadv.adn6028

**Published:** 2024-05-17

**Authors:** Rana Darweesh, Rajesh Kumar Yadav, Elior Adler, Michal Poplinger, Adi Levi, Jea-Jung Lee, Amir Leshem, Ashwin Ramasubramaniam, Fengnian Xia, Doron Naveh

**Affiliations:** ^1^ Faculty of Engineering, Bar-Ilan University, 52900 Ramat-Gan, Israel.; ^2^ Institue of Nanotechnology and Advanced Materials, Bar-Ilan University, 52900 Ramat-Gan, Israel.; ^3^ Department of Electrical Engineering, Yale University, New Haven, CT 06511, USA.; ^4^ Department of Mechanical and Industrial Engineering, University of Massachusetts, Amherst, MA 01003, USA.; ^5^ Materials Science and Engineering Graduate Program, University of Massachusetts, Amherst, MA 01003, USA.

## Abstract

Computational spectrometry is an emerging field that uses photodetection in conjunction with numerical algorithms for spectroscopic measurements. Compact single photodetectors made from layered materials are particularly attractive since they eliminate the need for bulky mechanical and optical components used in traditional spectrometers and can easily be engineered as heterostructures to optimize device performance. However, such photodetectors are typically nonlinear devices, which adds complexity to extracting optical spectra from their response. Here, we train an artificial neural network to recover the full nonlinear spectral photoresponse of a single GeSe-InSe p-n heterojunction device. The device has a spectral range of 400 to 1100 nm, a small footprint of ~25 × 25 square micrometers, and a mean reconstruction error of 2 × 10^−4^ for the power spectrum at 0.35 nanometers. Using our device, we demonstrate a solution to metamerism, an apparent matching of colors with different power spectral distributions, which is a fundamental problem in optical imaging.

## Teaser

A GeSe-InSe semiconductor device, enabled by an artificial neural network, provides accurate high-performance spectrometry.

## INTRODUCTION

**Fig. 1.**
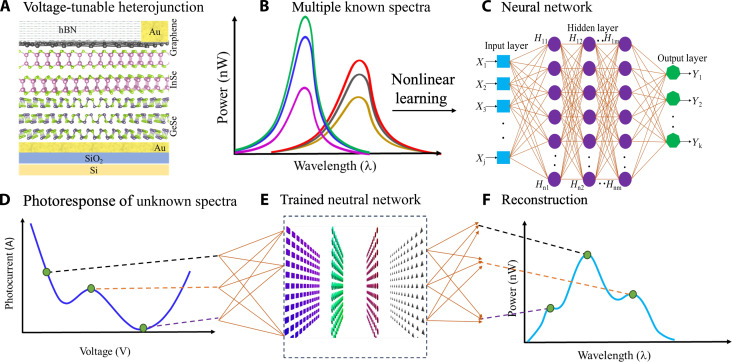
Illustration of the nonlinear learning and reconstruction process. (**A**) Schematic atomistic representation of vertical InSe/GeSe device (**B**) that is exposed to known power-modulated spectra, (**C**) for training an artificial neural network that captures the device’s nonlinear response. (**D**) A measured photocurrent vector of an unknown spectrum (**E**) is then analyzed with the trained neural network, (**F**) enabling the reconstruction of the unknown spectrum.

Optical sensing, the measurement of the properties of light such as its spectrum, polarization, and power, is central to several fields of science and technology (*1*–*3*). Accurate optical spectrometers with analytical calibration and resolution are typically expensive tabletop machines containing moving optical components (*4*, *5*). In contrast, computational optical spectroscopy (*6*–*8*) and sensing (*9*, *10*), which entails the use of an algorithm and either a single on-chip tunable detector or an array of detectors to replace the optical components in conventional sensing instruments, allows for spectral (*7*) and polarization (*11*) measurements in an efficient and compact manner. The operational principle of a computational spectrometer based on a single tunable device exploits the electrical tunability of the photodetector (*6*, *7*), e.g., via voltage bias, for collecting the output photocurrent within a high-dimensional vector space defined by the number of variable input conditions. This process is termed an encoding process and, so far, it has been applied only to linear detectors since the nonlinear problem was considered intractable. Linear computational detectors exploit the relation $I_{\text{ph}}\left(V\right)=\int_{\lambda_{1}}^{\lambda_{2}}R\left(V,\lambda \right)P\left(\lambda \right)d\lambda$ , where *R* is the responsivity, P(λ) is the spectral power density to be measured, *I*_ph_(*V*) is the photocurrent, and λ is the wavelength (*7*, *12*). The voltage-dependent responsivity matrix *R*(*V*; λ) (*6*, *7*, *13*) operates as a transformation operator that maps the measured photocurrent *I*_ph_(*V*) onto the spectrum  P(λ). These linear relations between the responsivity, current, and spectrum allow for training a transformation matrix that connects the measured photocurrent to the unknown spectrum, via linear regression techniques (*6*, *7*, *13*). However, most semiconductor devices can also operate in the nonlinear regime (*14*, *15*). For example, diodes, field-effect transistors, and bipolar transistors often display a nonlinear response that is considered disadvantageous for high-fidelity analog and digital communication systems (*16*, *17*) and optical spectrometers (*18*). In the case of nonlinear photoresponse, the existence of a mapping between the photocurrent and spectrum is not guaranteed, and if such a mapping exists, it must rely on a larger parameter space to account for the nonlinearity. In this work, we use a voltage-tunable heterojunction device of p-GeSe/n-InSe (see Fig. 1A) that is amenable to tuning both the spectral response and its higher-order nonlinearities. To resolve the complex mapping transformation between the spectrum and photocurrent, we encode the nonlinear response of the device by training an artificial neural network (ANN) (Fig. 1, B and C) that captures the device’s response as a function of bias voltage and spectrum. Unknown spectra can then be analyzed by decoding the voltage-dependent photocurrent response of the device (Fig. 1D) and performing the inverse mapping via the trained ANN (Fig. 1E) to reconstruct the power spectrum (Fig. 1F).

## RESULTS

**Fig. 2.**
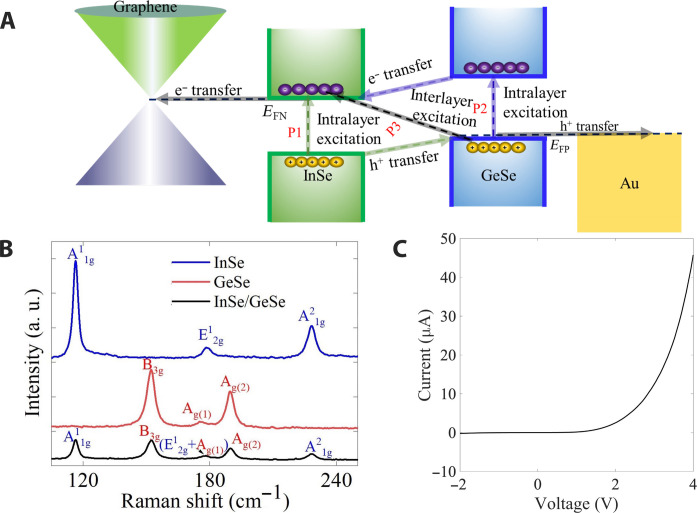
Schematic and Raman spectra of the device. (**A**) A schematic depicting the voltage-adjustable band alignment of InSe/GeSe, the main photoexcitation (P1, P2, and P3), and charge transfer routes driving the tunable photoconductivity (**B**) and the corresponding Raman spectra of InSe, GeSe, and InSe/GeSe, respectively. (**C**) The device current-voltage output curve confirms the formation of a p-n junction.

The voltage-tunable InSe/GeSe heterojunction device (Fig. 2A) consists of a stack of ~4 layers of p-type GeSe and ~7 layers of n-type InSe, the GeSe side is in contact with a bottom gold electrode that is also an optical mirror that increases the total cross section of the junction and the InSe side is in contact with transparent graphene electrode, while the entire device is encapsulated in hBN (Fig. 1A and fig. S1). The transparent single-layer graphene top contact is placed to allow optimal transmission of light to the junction. Utilization of the almost transparent graphene electrode not only enhances the transmission of light but also allows for direct, efficient photocarrier collection along the vertical direction. The Raman spectrum corresponding to this structure is presented in Fig. 2B (figs. S2 and S3), and the current-voltage output curves confirm the formation of a p-n junction (Fig. 2C and fig. S4; also see fig. S5 for results of a lateral device as a control experiment). While the principle of voltage-tunable band alignment and spectral response in two-dimensional (2D) heterostructures is known (*6*, *13*), the nonlinear response of the GeSe-InSe device is not yet understood. Previous reports on the nonlinear optical response of these materials have dealt primarily with the second-harmonic generation, originating from their non-centrosymmetric crystal structure (*19*–*21*). In the present case, the tunable nonlinear response of the heterostructure device arises from interfacial charge transfer at the p-n junction.

The GeSe-InSe system is a type II heterojunction, where InSe and GeSe are direct bandgap semiconductors with a single energy valley at the zone center (*22*), rendering the valence band edge centered on GeSe and the conduction band edge on the InSe side of the junction. Because of their direct bandgap nature, both InSe and GeSe strongly absorb the light. The tunable photodetection mechanism of the vertical p-GeSe/n-InSe device as described in Fig. 2A is composed of at least three substantial photoconduction channels: Two channels are related to intralayer excitations that are followed by minority charge transfer across the junction, as indicated in Fig. 2A, and labeled as P1 and P2 for the two processes. In addition, there is an interlayer optical excitation (marked as P3 in Fig. 2A) that is followed by a combined minority charge transfer from both sides of the junction. The applied voltage tunes the band alignment and thus strongly modifies the charge transfer rates across the heterojunction, leading to a nonlinear voltage-tunable response.

**Fig. 3.**
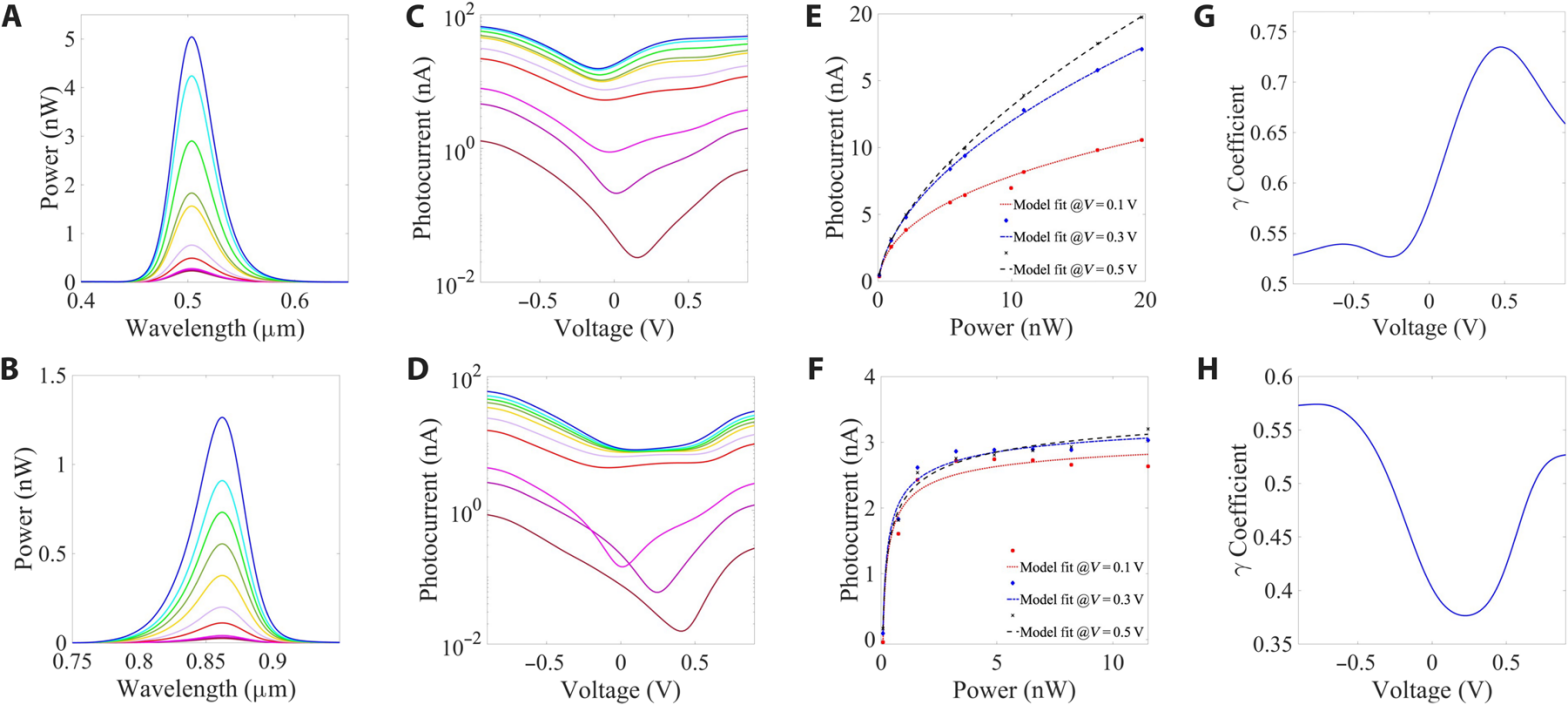
The training set and its nonlinear fitting. (**A** and **B**) Spectral power density of light-emitting diode (LED) sources from the nonlinear training set, (**C** and **D**) corresponding to the voltage-dependent photocurrent, (**E** and **F**) with a nonlinear saturation depicted from the measured photocurrents at fixed voltages of 0.1, 0.3, and 0.5 V [black cross, blue diamond, and red dot, respectively, (**G** and **H**) fitted to dash, dash-dot, and dotted lines] that are translated to a voltage-dependent nonlinear coefficient.

When the bias voltage is applied to this heterojunction, the built-in potential is modified as well as the optical polarizability associated with the observed voltage and nonlinear spectral response (Fig. 3). A general model describing the nonlinear response of the device may be written as

$$I_{V}=\sum_{\lambda }R_{V,\lambda }P_{\lambda }^{\gamma_{\left(V,\lambda \right)}},\left\{ \begin{array}{c} \lambda \in \left[\lambda_{1}\ldots \lambda_{N}\right]\\ V\in \left[V_{1}\ldots V_{M}\right] \end{array}\right.$$ (1)

where the nonlinear coefficients γ(*V*, λ) are unknown and span a high-dimensional *N* × *M* parameter space, *I_V_* is the photocurrent at voltage *V*, *R*_*V*,λ_ is the responsivity at voltage *V* and wavelength λ, and *P*_λ_ is the component of the power spectrum at wavelength λ. The dependence of our device’s nonlinear response on the applied voltage and spectrum is evaluated from the measurements in Fig. 3.

The InSe-GeSe heterojunction device was illuminated by seven different light sources, with each source being power-modulated at 10 different intensities (Fig. 3, A and B) while covering the spectral range of 400 to 1100 nm, and the resulting voltage-dependent photocurrents recorded, as shown in Fig. 3 (C and D). From these power-dependent *I*_ph_(*V*) data, the photocurrent-power relation is retrieved for each fixed voltage as shown in Fig. 3 (E and F) and is then fit to a saturating nonlinear model, *I*_ph_ = *RP*^γ^, where the total power is defined by $P=\int_{0}^{\infty }P\left(\lambda \right)d\lambda$ . Eventually, the voltage-dependent nonlinear coefficients, γ(*V*, λ), are evaluated for each spectrum (Fig. 3, A and B) and, as seen from Fig. 3 (G and H), depend strongly on both the spectrum and voltage. In this work, we measured the device response for *N* = 4000 wavelengths and *M* = 101 voltages, after which we interpolated the data to *N* = *M* = 2000 points (see Materials and Methods for details). This leads to a set of *D_NM_* = 4 × 10^6^ nonlinear coefficients, according to the model in Eq. 1. This large number of free independent parameters adds significant computational complexity as compared to previously studied linear response models (*6*, *13*, *23*–*28*)

**Fig. 4.**
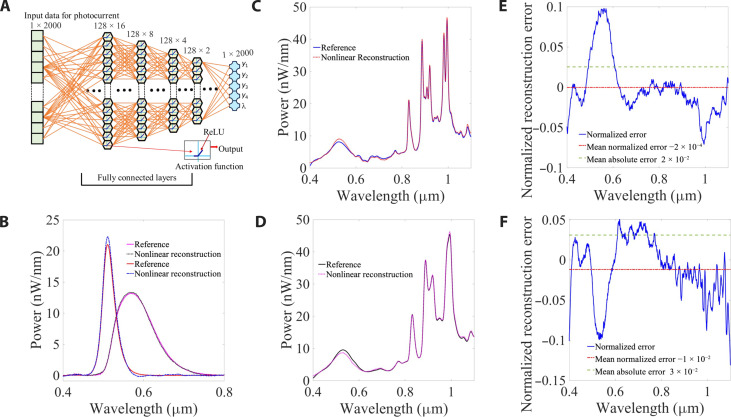
Nonlinear reconstruction of power spectra. (**A**) Schematic of the fully connected MLP network trained in the encoding process, comprising four hidden layers with a ReLU activation function and input/output vectors of dimension 2000 × 1. (**B**) The reference power spectral density (pink and red lines) and corresponding reconstruction (black dotted and blue dash lines) of two LED light sources. (**C**) The reference spectrum (blue line) and reconstruction (red dotted line) of a color-printed transparency, (**D**) and the same sample reconstructed at a resolution of 1000 × 1 input/output vectors. (**E** and **F**) The reconstruction error corresponding to (C) and (D) (blue line), the error mean (red dashed line), and the absolute error mean (green dot-dashed line), respectively.

**Fig. 5.**
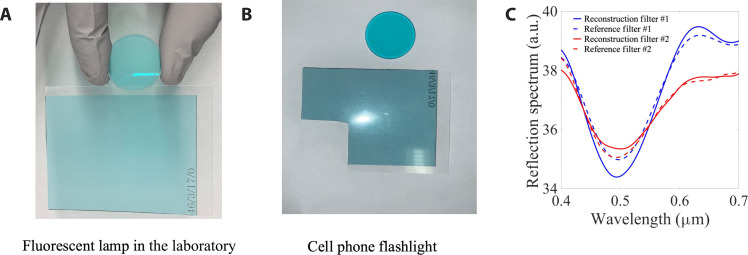
Metamaterials experiment. A photo of two filters of different colors taken under illumination by (**A**) a fluorescent lamp and (**B**) a cell phone flashlight. (**C**) The reflection spectra corresponding to the two filters as recorded with a reference commercial spectrometer (dashed line) and with our nonlinear spectrometer (solid line).

In the encoding process, pairs of known spectrum and corresponding measured photocurrent vectors were used for training a multilayer perceptron (MLP) neural network with four fully connected hidden layers. The hyperparameter space of the MLP was optimized using 128 batches, consisting of four layers with 2048, 1024, 512, and 128 neurons, and a ReLU activation function: *f*(*x*) = max {0, *x*} (Fig. 4A). The dataset was split randomly into a training set (80% of all data) and test set (20%) (see Materials and Methods and Supplementary Materials for further details on the training set and procedure). The reconstruction of the power spectrum of two light-emitting diode (LED) sources is demonstrated in Fig. 4B, showing a relatively low deviation of up to ~5 pW/nm—as compared to a benchtop reference spectrometer (see Materials and Methods). This sensitivity results from the training set of Fig. 3 (A and B) and fig. S6, spanning three degrees of order in the power. Furthermore, the resolution of the reconstructed power spectra was evaluated with respect to the dimension of the input/output vectors (photocurrent and spectrum) by reducing the dimension of the power spectrum vectors from 2000 × 1 to 1000 × 1. Figure 4C shows the measured and reconstructed spectra of color-printed polymer transparency (such as the ones shown in Fig. 5), sampled with 2000 points over the spectral range of 400 to 1100 nm. The lower-resolution spectrum of dimension 1000 × 1 is shown in Fig. 4D. The low-resolution spectrum was decoded with a similar MLP with hidden layers of 1024, 512, 256, and 128 neurons. Even upon decreasing the vector size by a factor of two, the power calibration (and dynamic range) of the spectrometer is maintained while the spectral resolution deteriorates, as observed from the broadening and loss of spectral details in Fig. 4D. Here, the spectral resolution is not only defined directly by the reconstruction of the computational spectrometer but also based on the resolution of the reference signal that was measured with a tabletop spectrometer. The quality of the computational spectrometer is defined by the reconstruction error relative to the reference signal used to test and match its performance. The normalized reconstruction error is defined as $E=\frac{P_{\text{ref}}-P_{\text{rec}}}{P}\text{∆}\lambda$ , where ∆λ is the spectral range, *P* is the total power, and $P_{\text{ref}}$ and $P_{\text{rec}}$ are the reference and reconstructed power spectra, respectively. The error is shown in Fig. 4 (E and F) for the high- and low-resolution cases presented in Fig. 4 (C and D), respectively. The mean value of the normalized error and of the absolute error (dash lines) are −2 × 10^−4^ and 2 × 10^2^, respectively for the high-resolution case of Fig. 4E and −10^−2^ and 3 × 10^−2^, respectively, for the lower resolution of Fig. 4F. Clearly, the reconstructed spectra follow the reference, as evident from these small errors. The unique working mechanism of our device leverages the tunable nonlinear spectral response of GeSe-InSe heterojunction, and using ANN, we generate a solution to a large set of nonlinear equations that depends on ~10^6^ nonlinear parameters—a problem that is considered a hard mathematical problem. With that, we obtain an accurate performance of the compact spectrometer with ~5 pW/nm power sensitivity, 0.35-nm wavelength resolution, and 400- to 1100-nm spectral range. Moreover, this concept holds promise for future development of 2D/3D diodes or heterojunctions (*29*, *30*) and for wafer scale incorporation of 2D materials, toward spectroscopic arrays integration (*31*).

## DISCUSSION

The ability to optically inspect objects with high spectral resolution within the visible–to–near-infrared range using a simple portable device has many potential applications in our daily lives. One such common example is the objectivity of colors in vision and imaging, and their dependence upon illumination, known as metamerism. In metamerism, two objects of different color can appear to have the same color or, alternatively, the same object can appear different under varying illumination. The two different filters in Fig. 5A appear indistinguishable under fluorescent ambient light. However, with a cell phone flashlight, the difference between these filters becomes evident (Fig. 5B).

With our compact nonlinear spectrometer, the reflection spectra of the two filters show a clear difference, reproduced both by our nonlinear spectrometer and the commercial spectrometer (Fig. 5C). This provides a quantitative measure of the true color of the filters, independent of the lighting conditions.

In conclusion, we have demonstrated a power-calibrated spectrometer based on a single, voltage-tunable GeSe-InSe p-n heterojunction device. Using ANNs to decode the nonlinear photocurrent response of this device allows us to achieve high-resolution spectral measurements over the entire visible-to-NIR range of the optical spectrum. Specifically, with a small device footprint of ~25 × 25 μm^2^ and a trained ANN, we were able to reconstruct complex power spectra within the spectral range of 400 to 1100 nm with accuracy better than 5 pW/nm and a spectral resolution of 0.35 nm. Our results pave the way for expanding the role of computational spectroscopy as a viable alternative to traditional optical spectrometry, potentially leading to single-element, on-chip spectrometers for rapid and inexpensive optical sensing.

## MATERIALS AND METHODS

### Device fabrication

The following steps were taken to fabricate an encapsulated hBN/Graphene/InSe/GeSe vdW heterojunction using the dry transfer method inside a glovebox to prevent contamination and degradation of the exfoliated samples. First, Si/SiO_2_ substrates were cleaned with deionized water, acetone, and isopropanol in succession, and then dried with nitrogen gas. Mechanical exfoliation was used to obtain the desired thickness of hBN, graphene, InSe, and GeSe flakes from their parental crystals on top of Si/SiO_2_ substrates. Next, the dry-transfer technique was used to encapsulate the vertical hetero-junction with hBN, creating a vertical hBN/Gr/InSe/GeSe heterostructure. The successive layers were picked up from a 285-nm Si/SiO_2_ wafer using a polycarbonate (PC) membrane, starting with the top layer of hBN, followed by the graphene flake, InSe, and lastly, the bottom layer of GeSe. The entire stack was deposited on a clean pre-patterned back electrode (Ti/Au:5/50 nm) at 150°C, and the PC membrane was then dissolved using chloroform.

### Sample thickness measurements

Samples of thin flakes (not the ones used in devices) exfoliated on 300-nm Si/SiO_2_ substrates were measured by atomic force microscopy (AFM) to calibrate their thicknesses to their optical contrast. As the actual devices were fabricated fully in a glovebox, the number of InSe and GeSe layers in the heterostructure was determined by the optical contrast calibration, further complemented by Raman spectroscopy (*32*). For InSe, the obtained Raman modes at 116.22, 178.49, and 227.90 cm^−1^ correspond to the characteristic modes (A^1^_1g_, E^1^_2g_, and A^2^_1g_, respectively) reported for seven layers of InSe (*33*, *34*). For GeSe, the obtained Raman modes at 152.3483, 175.2849, and 189.648 cm^−1^ correspond to the characteristic modes B_3g_, A_g (1)_ and A_g (2),_ respectively. The difference between A_g (2)_ and B_3g_ modes is 37.2997 cm^−1^, in agreement with the reported difference for four layers of GeSe (*35*, *36*). Furthermore, our previous thickness-dependent AFM measurements also confirm that the error falls within ± one layer.

### Photoresponse characterization

All measurements of photocurrent as a function of bias voltage were performed at room temperature (25° ± 0.1°C) under vacuum conditions at ~10^−5^ torr. The photocurrent was measured with incident light modulated by a mechanical chopper at a frequency of 1 kHz, and with a low noise current preamplifier (Femto, DLPCA-200) and lock-in amplifier (Model SR830). In this photocurrent measurement, the heterojunction was illuminated by seven LEDs and a Laser-Driven Light Source (LDLS) as a white-light source combined with a set of bandpass filters and with transparency printed filters (see the Supplementary Materials for details). The reference spectrum of each light source was measured with a Thermo Fisher Scientific Nicolet iS50R Fourier Transform Infrared spectrometer connected to an external silicon detector (Thorlabs, FDS100), and the spectra were normalized to the silicon detector’s calibrated responsivity.

### Computational spectrum reconstruction

The dataset (see the Supplementary Materials) comprising (i) 10 power spectra acquired from seven LED light sources and (ii) LDLS with filter sets as follows: (a) a bandpass filter set (at width of 25 nm) in the spectral range of 400 to 1100 nm; (b) a bandpass filter set (at width of 40 nm) in the spectral range of 400 to 1100 nm; and (c) a set of homemade filters produced by printing transparencies. All filter sets were spectrally characterized. Overall, 50 filters were used with the white light source. The data were split randomly into training (80%) and testing (20%) sets. The reconstruction code used TensorFlow, sklearn, numpy, and scipy software packages.
