# Supplementary material for: Nonlinear self-calibrated spectrometer with single GeSe-InSe heterojunction device
Source: Sci Adv. 2024 May 17;10(20):eadn6028. doi: 10.1126/sciadv.adn6028 (PMC11100572; doi:10.1126/sciadv.adn6028)
Supplement: Supplementary file 1 — Figs. S1 to S8 [file sciadv.adn6028_sm.pdf]

Science Advances
AAAS

Supplementary Materials for

Nonlinear self-calibrated spectrometer with single GeSe-InSe heterojunction device

Rana Darweesh *et al.*

Corresponding author: Doron Naveh, doron.naveh@biu.ac.il

Sci. Adv. **10**, eadn6028 (2024)
DOI: 10.1126/sciadv.adn6028

This PDF file includes:

Figs. S1 to S8

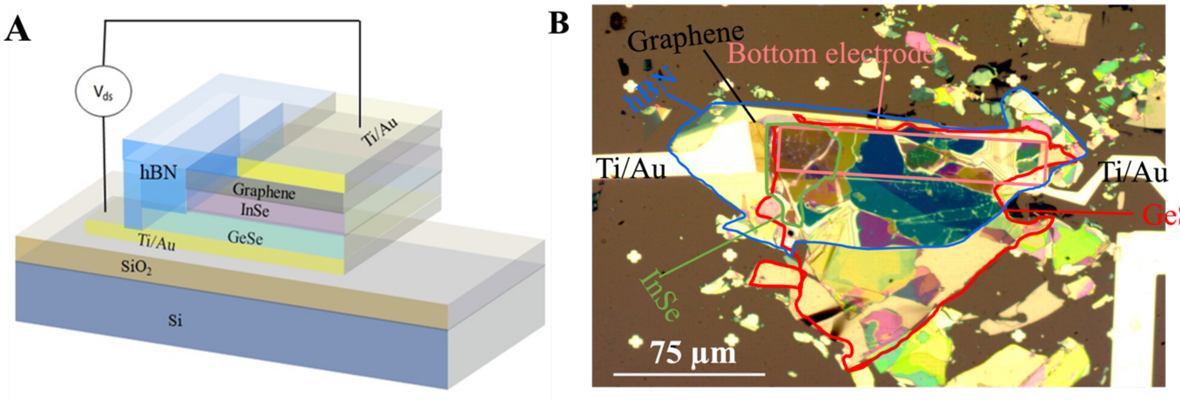

Fig. S1. Schematic and optical images of a vertical heterostructure device: (A) Depiction of the vertical heterostructure composed of hexagonal boron nitride (hBN), graphene, indium selenide (InSe), and germanium selenide (GeSe). This schematic offers a top view representation of the layered arrangement. **(B)** High-resolution optical image of the vertical heterostructure. The fabrication process adhered to the detailed procedures outlined in the Methods section, ensuring precise layer alignment and material deposition.

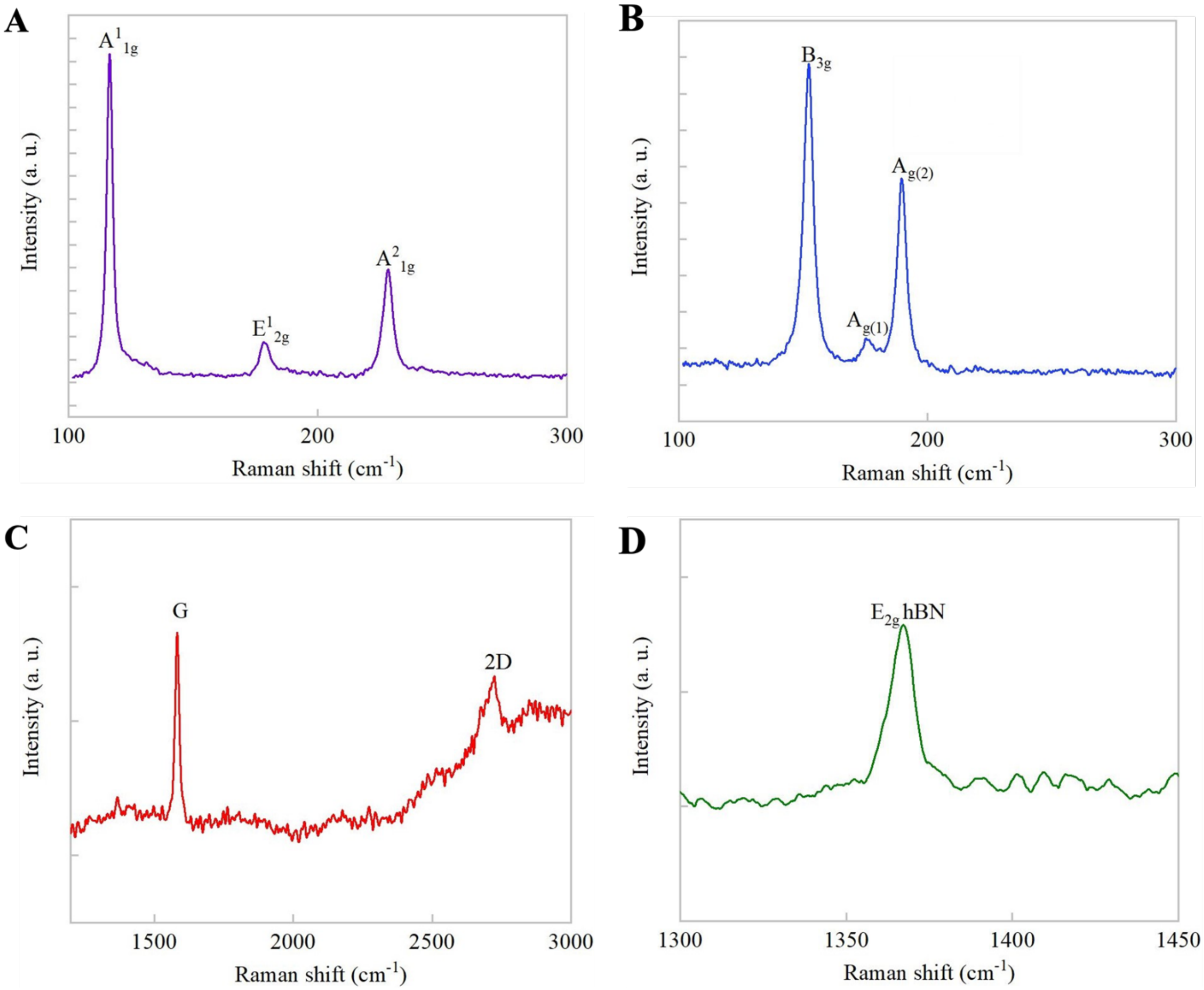

Fig. S2. Raman spectra of a heterostructure device after stacking. Individual (non-overlapping) flakes of **(A)** InSe, **(B)** GeSe, **(C)** Graphene, and **(D)** hBN.

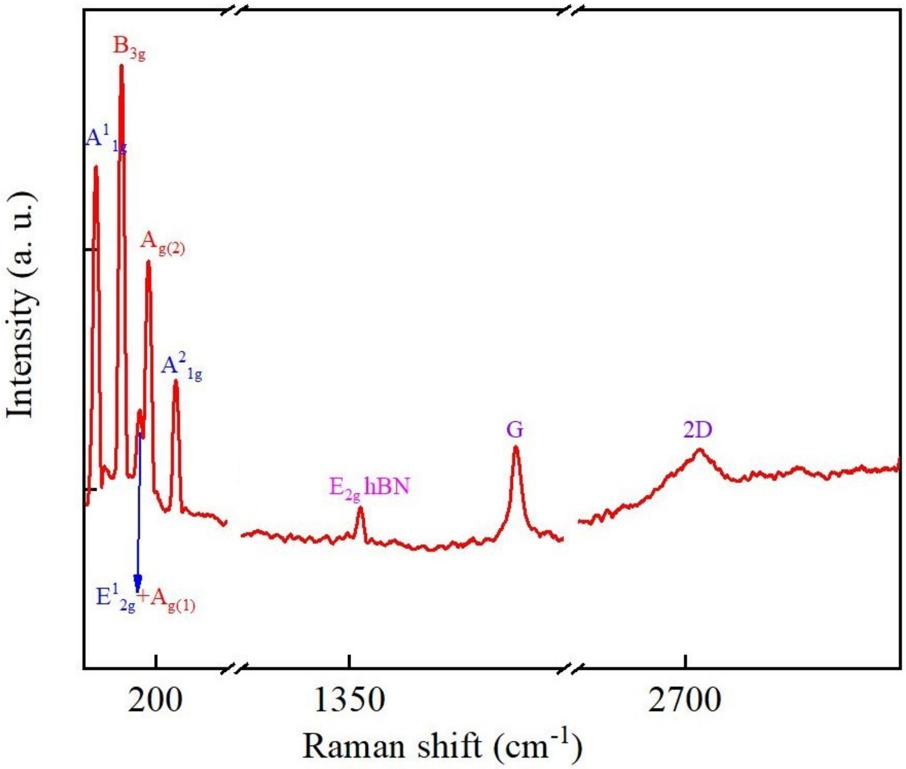

Fig. S3. The Raman spectrum of the heterostructure comprising the spectrometer device exhibits a combination of vibrational modes, including G and 2D modes of graphene, E_{2g} modes of hBN, A^1_{1g}, E^1_{2g} and A^2_{1g} modes of InSe and B_{3g}, $A_{g\,(1)}$ and $A_{g\,(2)}$ modes of InSe.

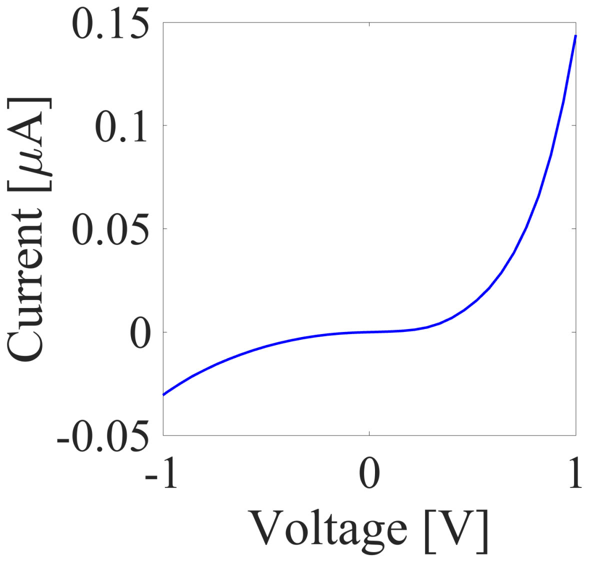

Fig. S4. An enlarged view of the device current-voltage output curve, measured over the range of ±1V, which appears nominally flat in Fig. 2C.

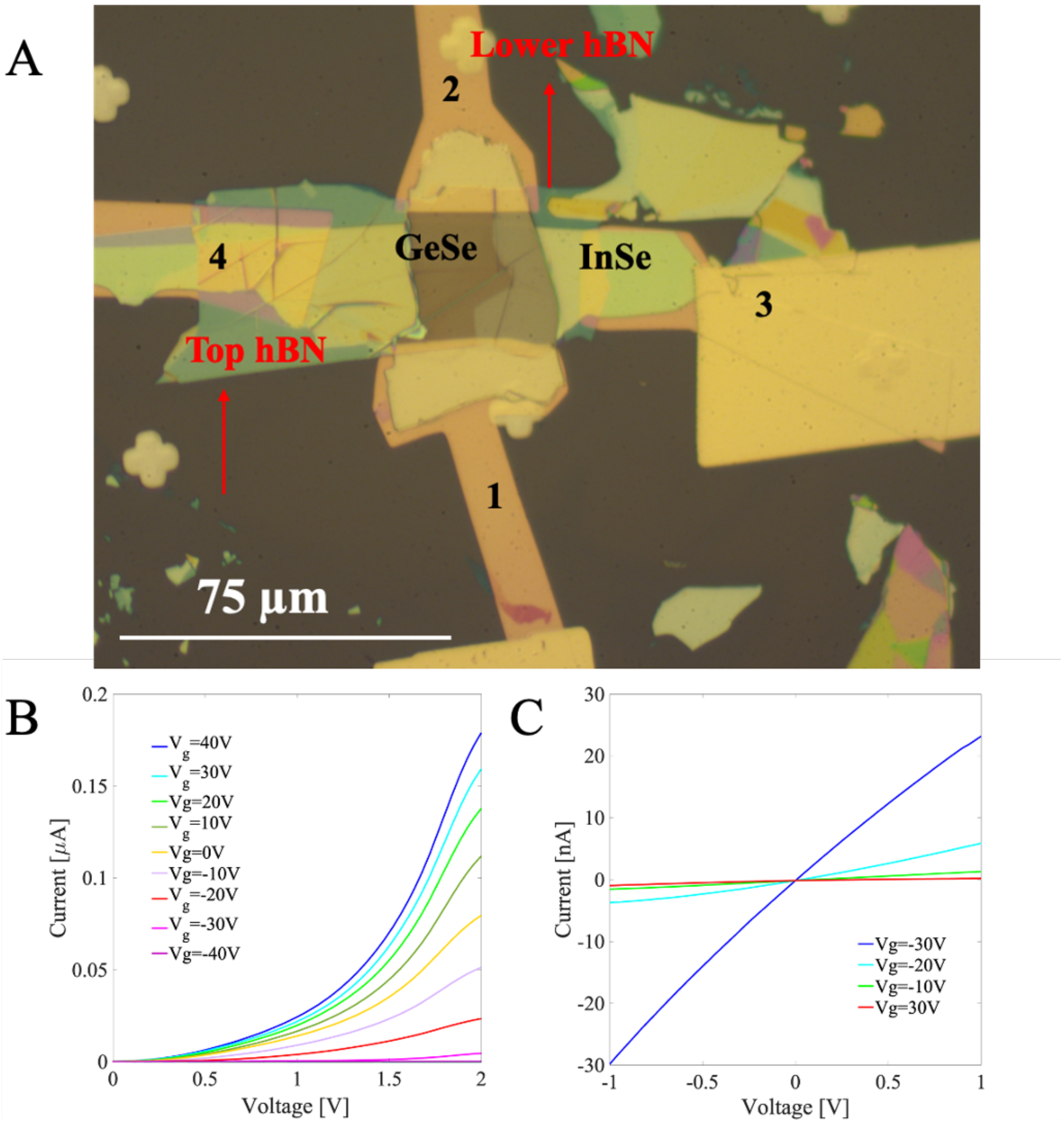

Fig. S5. Fabricated lateral device and its current vs. voltage measurements. (A) Optical micrograph of a lateral hBN/InSe/GeSe heterostructure. Contacts 1 and 2 were electrically connected to the GeSe flake, while contacts 3 and 4 were linked to the InSe flake. **(B)** The I-V curves of a p-n GeSe/InSe lateral junction (contact 1 to 3) sampled with applied gate voltage bias. **(C)** The current-voltage curve of GeSe (contacts 3 and 4) showing linear behavior, presented for both negative and positive bias voltages.

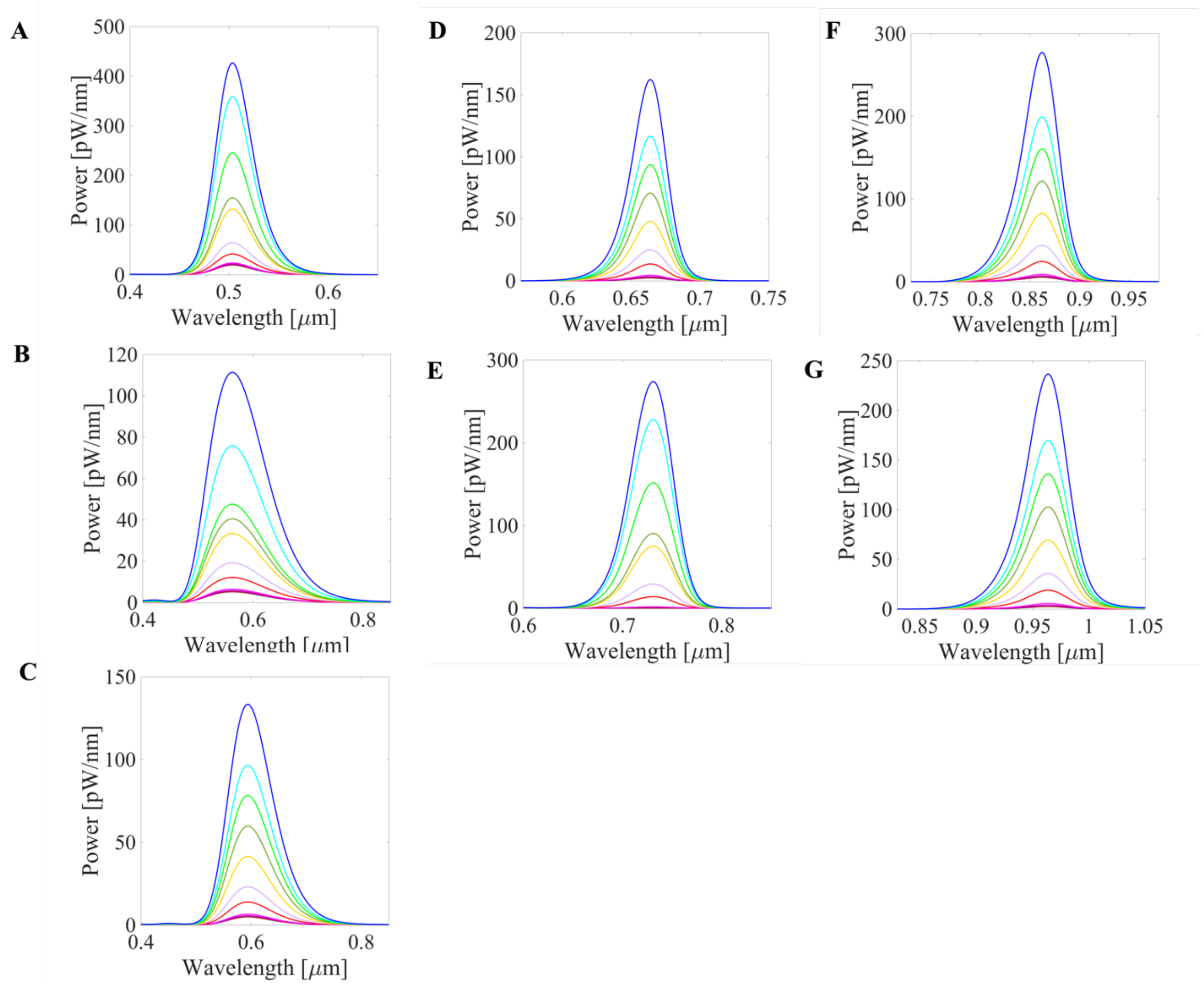

Fig. S6. Dataset of the LED spectra used to train the ANN. The reference spectra of seven LEDs covering the spectral range of the device. The LEDs are centered at wavelengths of **(A)** 505 nm, **(B)** 565 nm, **(C)** 595 nm, **(D)** 660 nm, **(E)** 730 nm, **(F)** 850 nm, and **(G)** 940 nm. Each LED was measured at ten intensities, highest in blue to lowest in brown.

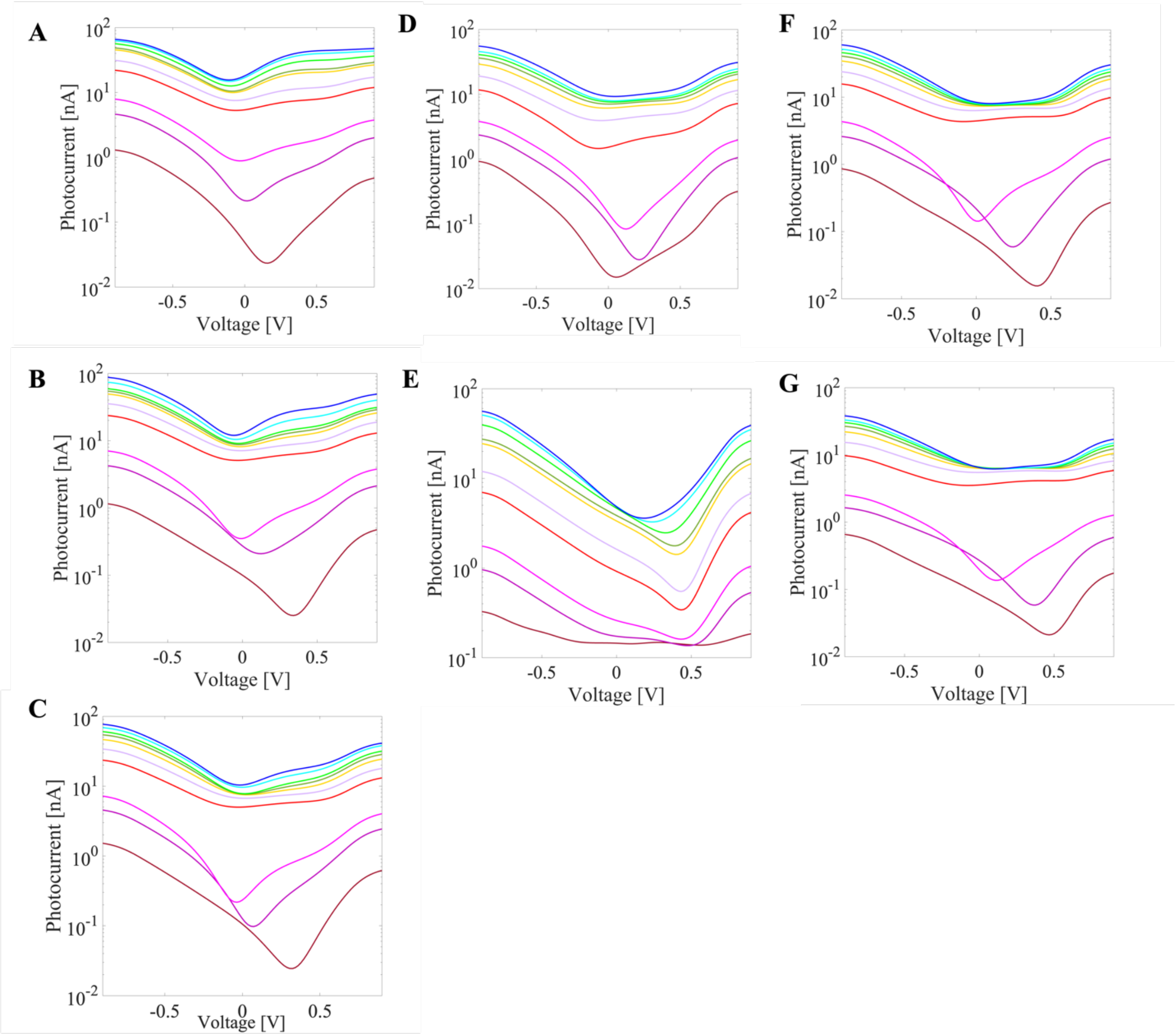

Fig. S7. Dataset of the LED photocurrents vs. voltage used to train the ANN. Photocurrent as function of voltage measured for seven LEDs covering the spectral range of the device. The LEDs are centered at wavelengths of **(A)** 505 nm, **(B)** 565 nm, **(C)** 595 nm, **(D)** 660 nm**, (E)** 730 nm, **(F)** 850 nm, and **(G)** 940 nm. Each LED was measured at ten intensities, highest in blue to lowest in brown.

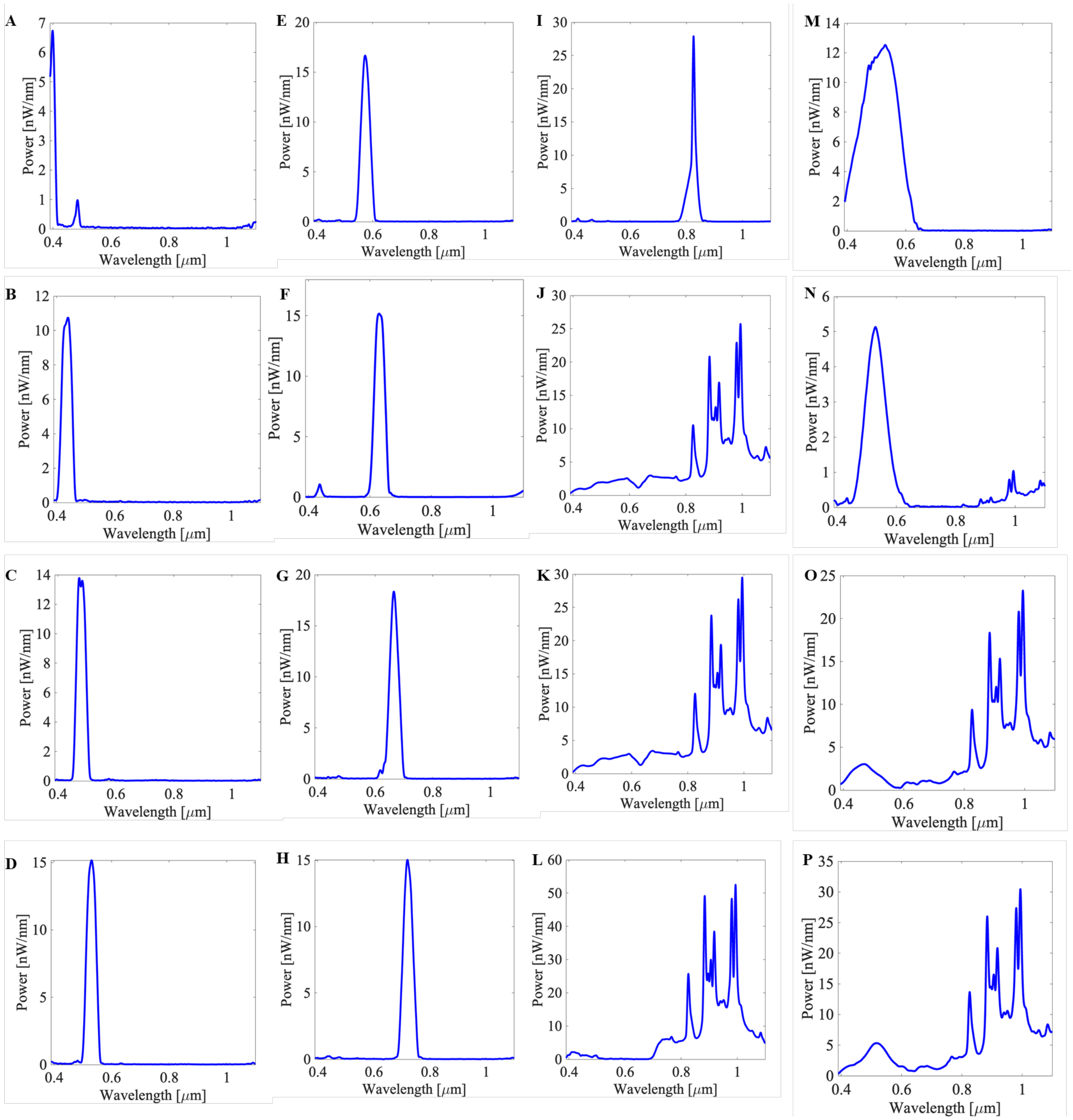

Fig. S8. Dataset of the spectra of Laser Driven Light Source (LDLS) with various filters used to train the ANN. The spectra were measured using (Thorlabs) bandpass spectral filters with Full Width at Half Maximum (FWHM) of 40 nm, depict wavelengths centered at **(A)** 400 **(B)** 450 **(C)** 500 **(D)** 550 **(E)** 600 **(F)** 650 **(G)** 700 **(H)** 750 **(I)** 850 nm. Additionally, spectral data were acquired utilizing (Thorlabs) bandpass filters of **(J)** DG10-600-B **(K)** DG10-1500-B **(L)** FGB25 **(M)** FGB39 **(N)** FGV9. Furthermore, spectral measurements also incorporated in house color printed polymer filters with color codes **(O)** 97_100_15_40 and **(P)** 100_0_76_0.
